# Leishmaniasis Vaccine: Where are We Today?

**DOI:** 10.4103/0974-777X.62881

**Published:** 2010

**Authors:** Lukasz Kedzierski

**Affiliations:** *Infection and Immunity Division, Walter+Eliza Hall Institute of Medical Research, Melbourne, Australia*

**Keywords:** Immune response, Leishmania, Vaccine

## Abstract

Leishmaniasis is a disease that ranges in severity from skin lesions to serious disfigurement and fatal systemic infection. WHO has classified the disease as emerging and uncontrolled and estimates that the infection results in two million new cases a year. There are 12 million people currently infected worldwide, and leishmaniasis threatens 350 million people in 88 countries. Current treatment is based on chemotherapy, which relies on a handful of drugs with serious limitations such as high cost, toxicity, difficult route of administration and lack of efficacy in endemic areas. Vaccination remains the best hope for control of all forms of the disease, and the development of a safe, effective and affordable antileishmanial vaccine is a critical global public-health priority. Extensive evidence from studies in animal models indicates that solid protection can be achieved by immunization with defined subunit vaccines or live-attenuated strains of *Leishmania*. However, to date, no such vaccine is available despite substantial efforts by many laboratories. The major impediment in vaccine design is the translation of data from animal models to human disease, and the transition from the laboratory to the field. Furthermore, a thorough understanding of protective immune responses and generation and maintenance of the immunological memory, the most important and least-studied aspect of antiparasitic vaccine development, during *Leishmania* infection is needed. This review focuses on recent findings in antileishmania vaccine field and highlights current difficulties facing vaccine development and implementation.

## INTRODUCTION

*Leishmania* are protozoan parasites shuttling between sand fly vector where they multiply as free promastigotes in the gut lumen, and mammalian host where they proliferate as obligatory intracellular amastigotes in the mononuclear phagocytes.[1] *Leishmania* parasites are responsible for a family of diseases, collectively known as leishmaniases, with discrete clinical features ranging from cutaneous lesions to a fatal systemic disease. Leishmaniasis is prevalent in Africa, Latin America, Asia, the Mediterranean basin and the Middle East, and recently has been identified in East Timor,[2] Thailand[3] and in kangaroos in Australia.[4] Leishmaniasis has been classified as one of the most neglected diseases, and the estimated disease burden places it second in mortality and fourth in morbidity among the tropical infections.[5] For many years, the public health impact of leishmaniasis has been underestimated, as a substantial number of cases were never recorded. The expansion of leishmaniasis and the sharp rise in prevalence is related to environmental changes and migration of non-immune people to endemic areas.[6] The former, in particular, has the potential to expand the geographic span of the vector, thus increasing *Leishmania* transmission to previously unaffected areas.[7] More recently, an increase in the overlapping of HIV infection and visceral leishmaniasis has been observed, especially in intravenous drug users in South-Western Europe and Brazil.[8] The situation might be much worse in Africa and Asia where the prevalence and detection of HIV and *Leishmania* co-infections is still largely underestimated.

Current treatment is based on chemotherapy, which relies on a handful of drugs with serious limitations such as high cost and toxicity, difficult route of administration and lack of efficacy in endemic areas. The pentavalent antimonials such as sodium stibogluconate and meglumine antimoniate have been recommended for the treatment of leishmaniasis for over 70 years. It is thus not surprising that resistance to this class of drug is increasing, and in some endemic areas their use is limited due to a lack of efficacy. Second line drugs used in the treatment of leishmaniasis include aromatic diamidines (Pentamidine) and amphotericin B, but similarly to the pentavalent antimonials, these drugs are toxic, with severe (sometimes life-threatening) side effects.[9] A development of a successful vaccine to prevent leishmaniasis has been a goal for almost a century, but currently no such vaccine exists. Extensive evidence from studies in animal models, mainly mice, indicates that solid protection can be achieved upon immunization with defined subunit vaccines (either protein or DNA) or heat-killed parasites, however, to date such vaccines have been disappointing when tested in field studies.[10] First attempts at vaccination, termed leishmanization, were based on the observation that following lesion healing an individual is refractory to reinfection. Initially, infectious lesion material, later replaced by cultured parasite inoculum, has been used to inoculate uninfected individuals. This method has been largely discontinued due to a range of reasons including quality control, parasite persistence, emergence of HIV and ethical reasons, amongst the others. The first-generation vaccines based on killed parasites have replaced leishmanization, but this type of vaccines have shown poor efficacy in clinical trials.[11] Although the first generation vaccines still undergo evaluation, the focus is now on the second generation vaccines including genetically modified parasites, defined subunit vaccines or recombinant bacteria and viruses expressing leishmanial antigens.[10] So far, their efficacy in the field trials has not been reported. *Leishmania* vaccine development has proven to be a difficult and challenging task, which is mostly hampered by inadequate knowledge of parasite pathogenesis and the complexity of immune responses needed for protection. These aspects are of key importance in the vaccine development process.

## CLINICAL FEATURES AND IMMUNOLOGY OF LEISHMANIASIS

Leishmaniasis in humans is caused by several species of *Leishmania*, which lead to strikingly different pathological responses. The cutaneous form of the disease (CL) accounts for more than 50% of new cases of leishmaniasis. It results in formation of skin ulcers at the site of the sand fly bite, usually on exposed parts of the body. The disease is usually self-limiting, but the time to lesion resolution varies between species and between individuals. Some species are also noted for causing non-healing cutaneous disease. Vast majority of cases (90%) occurs in Afghanistan, Middle East and South America. Related diseases include diffuse cutaneous leishmaniasis (DCL) that occurs in anergic hosts with poor immune responses, and mucocutaneous leishmaniasis (ML) characterized by the late development of metastatic lesions that can lead to destruction of the mucous membranes. Visceral leishmaniasis (VL), also known as *kala-azar*, is the most severe and often fatal syndrome. Visceral species such as *L. donovani, L. infantum* and *L. chagasi*, target visceral organs and result in the pentad of syndromes comprised of fever, weight loss, splenomegaly, hepatomegaly and anemia. Majority of cases occurs in India, Bangladesh, Nepal, Brazil and Sudan. Between 20 to 60% (depending on geographical location) of VL patients develop a syndrome known as post *kala-azar* dermal leishmaniasis (PKDL), which appears within a few years of the complete cure of VL. PKDL patients are considered a major source of parasites for new infections because of the large number of organisms in the skin accessible to sand fly bites.

Antileishmanial immunity is mediated via both innate (macrophages, neutrophils) and adaptive (B cells, T cells and DCs) immunity. Macrophages play a pivotal role in *Leishmania* infection. A successful treatment of all the forms of leishmaniasis depends on efficient elimination of parasites by activated macrophages. Paradoxically, *Leishmania* utilises their phagocytic function as a strategy for internalization and replication within the phagolysosomes.[12] Thus, macrophages act as both the host cells and effector cells that kill the parasites. Internalization of *Leishmania* by macrophages leads to the production of proinflammatory cytokines and parasite killing. A subversive activity of *Leishmania* parasites in this process is the inhibition of interleukin-12 (IL-12) production, which is necessary for the leishmanicial activity of macrophages,[13] as it leads to upregulation of inducible nitric oxide synthase (iNOS), nitric oxide (NO) and interferon gamma (IFN-γ). Production of cytokines results in the recruitment of other proinflammatory cells (neutrophils, mast cells and macrophages) to the site of infection. In particular, neutrophils are among the first cells recruited to the site of infection and are thought to participate in the containment of *Leishmania* parasites within an hour of infection.[14] Published data on the involvement of neutrophils in *Leishmania* infection are contradictory, indicating either their role in resistance to leishmaniasis or disease exacerbatory activities.[15] However, it has been shown that in the context of infection initiated by the bite of an infected sand fly, neutrophils are recruited to the site of infection and phagocytose parasites, a process that is vital for disease progression.[16] These findings suggest that neutrophils, apoptotic neutrophils in particular, are more likely to play a role in promoting disease progress, rather than resistance. An important component linking the innate and adaptive immune responses are dendritic cells (DC), which are recruited in response to production of mast cell-derived mediators and cytokine/chemokine release by macrophages and neutrophils.[12]

Amastigote uptake by DCs at the site of infection results in the upregulation of IL-12,[17] which is essential for parasite elimination, and for the effector functions of macrophages.[18] The ability of DCs to present antigens through the MHC I and II pathways leads to stimulation of *Leishmania* -specific CD4^+^ and CD8^+^ T cell responses[19] and is essential for acquired resistance against *Leishmania*. CD4^+^ T cells play an important role in antileishmanial immunity and disease outcome. Early experimental studies in a mouse model of cutaneous leishmaniasis, established a clear-cut dichotomy between Th1-mediated protection and Th2-mediated disease susceptibility. Failure to mount an efficient anti-*Leishmania* Th1 response was shown to cause progressive disease and absence of lesion resolution.[20] In resistant C57BL/6 mice, resolution of the disease is mediated as a consequence of IFN-γ release by Th1 cells and upregulation of NO in macrophages that harbor parasites.[21] Conversely, persistence of lesions in susceptible BALB/c mice is due to Th2-type CD4^+^ T cell differentiation and production of IL-4, which suppresses macrophage activation, resulting in parasite survival.[20] However, the Th1/Th2 dichotomy has been questioned in recent times since there is accumulating evidence that early IL-4 response might not be required to promote susceptibility and there are more complexities in the mechanisms responsible for acquired immunity.[19] Thus, the Th1/Th2 dichotomy as an indicator of resistance and susceptibility might be a generalization and is far more complex than what we currently know and understand.

The cytokine production and cytotoxic activity by CD8^+^ T cells also contribute to the disease outcome in *Leishmania* infection. Initially, CD8^+^ T cells were thought to play a role only during re-infection,[22] however, they were also shown to be crucial in controlling the primary infection by skewing the responses towards Th1-type.[23,24] Besides cytokine production, CD8^+^ T cells are also thought to participate in controlling the infection through cytotoxic mechanisms, such as granzyme and perforin production and Fas/FasL pathways, but these data are contradictory.[25] It is still not known what is the exact route of CD8^+^ T cell activation in leishmaniasis, since the parasites reside in a parasitophorous vacuole inside the host macrophages and it is not clear how these cells present antigen through MHC I.[26,27] The most likely mechanism is cross-presentation, which has been well documented for macrophages and DCs,[28,29] but has not yet been demonstrated in *Leishmania* infection. Therefore, the exact role of CD8^+^ T cells in *Leishmania* infection, including fine-specificities of CD8^+^ T cell epitopes and the route of their activation, remain to be elucidated.

The vast array of cytokines[9] and immune mechanisms involved in the immune response to *Leishmania* clearly highlight the complexity of the disease. The murine model of cutaneous leishmaniasis, which mimics many aspects of the human disease and from where majority of our current knowledge of *Leishmania* immunology is derived, has also been used as a tool for assessing vaccine efficacy. One caveat is the fact that the precise immune mechanisms underlying human cutaneous leishmaniasis are still not fully understood, and the responses necessary for protection by vaccination are not as clear as in the mouse model.[30] It appears that in humans, the outcome of disease is influenced by the balance between Th1 and Th2 type responses and is further affected by the host genetic factors, inoculum size and parasite strain. Despite these shortcomings, the mouse model of leishmaniasis has been extremely beneficial in testing vaccine candidates.

## OVERVIEW OF VACCINE DEVELOPMENT

Leishmaniasis is a disease that is most likely to be controlled by a successful vaccination program. The relatively uncomplicated leishmanial life cycle and the fact that recovery from a primary infection renders the host resistant to subsequent infections indicate that a vaccine is feasible. Evidence from studies in animal models, mainly mice, indicates that protection can be achieved upon immunization with various vaccine formulations, however, to date such vaccines have been disappointing when tested in the field.[10] Majority of experimental vaccines were tested against the cutaneous form of the disease in the mouse model. Although the demands for a VL vaccine are more complex than for a CL vaccine, it is believed that human VL trials will follow any successful CL immunization program. Whether the same vaccine will work against both forms of the disease remains to be seen. Presently, VL vaccination studies are hampered by the lack of a suitable animal model of disease. The best animal models are the natural combination of dogs and *L. infantum* or *L. chagasi*[31] and *L. donovani* in golden hamsters.[32] However, both models use outbred animals and suffer from lack of immunological reagents and assays needed for the dissection of immune responses.

To date, several different approaches to antileishmanial vaccine have been tested. First generation vaccines composed of whole killed parasites have been proposed as both prophylactic and therapeutic vaccines. The therapeutic application may be particularly important in cases of drug resistant refractory disease. In theory, these vaccines should be easy to produce at a low cost in endemic countries; however, standardization of cultured parasite-derived vaccines is a drawback in the way to their registration. In general, the whole-cell, killed vaccines have been rather poorly defined and variable in potency, hence they have rendered inconclusive results.[11] Nevertheless, the trials completed so far demonstrated their good safety profile, and despite poor prophylactic outcomes, showed encouraging results as therapeutic vaccines in South America and Sudan.

Most of the vaccine studies concentrate on the second generation vaccines consisting of recombinant proteins, poly-proteins, DNA vaccines or dendritic cells loaded with peptides derived from leishmanial antigens. A variety of different molecules has been tested to date, and these included antigens such as surface expressed glycoprotein leishmaniolysin (gp63) delivered by a plethora of immunization regimens, however, promising findings from animal models were overshadowed by mostly negative T cell responses in humans.[33] Another vaccine candidate has been a GPI-anchored membrane protein gp46 or Parasite Surface Antigen 2 (PSA-2), that belongs to a gene family present in all *Leishmania* species except *L. braziliensis.*[34] PSA-2 is involved in macrophage invasion through the interaction of its leucine rich repeats with complement receptor 3.[35] Immunization with the native polypeptides derived from promastigotes protected mice against infection,[36] but vaccination with a recombinant protein derived from either promastigotes or amastigotes protein showed lack of protective efficacy.[37] Similarly, DNA vaccination conferred protection in mice when used as either prophylactic[38] or therapeutic vaccines.[39] Another extensively tested antigen is the *Leishmania* homologue for receptors of activated C kinase (LACK) that is expressed throughout leishmanial life cycle.[40] Immunization with LACK appears to promote the expansion of IL-4 secreting T cells skewing the response towards detrimental Th2 responses,[41] however, susceptible BALB/c mice immunized with LACK had the ability to control a subsequent infection with *L. major*.[42] To date, the protective efficacy of LACK has been mainly demonstrated in the *L. major* model, and LACK failed to protect against visceral leishmaniasis.[43]

Several other antigens from different species have been tested in animal models. These include amastigote cysteine proteases (CP),[44] cysteine proteinase A2 and amastigote membrane proteins P4 and P8,[45] kinetoplastid membrane protein-11 (KMP-11),[46] amastigote LCR1,[47] hydrophilic acylated surface protein B1 (HASPB1),[48] leishmanial antigen ORFF,[49] acidic ribosomal protein P0,[50] paraflagellar rod protein 2 (PRP-2),[51] NH36, a main component of the fucose-mannose ligand,[52] and proteophosphoglycan (PPG).[53] In addition, molecules such as ATP synthase alpha chain, beta-tubulin and heat shock 70-related protein 1 precursor have been recently identified as novel vaccine candidates.[54]

To date, only one second generation vaccine, Leish-111f, has been assessed in clinical trials.[55] Leish-111f is a single polyprotein composed of three molecules fused; the *L. major* homologue of eukaryotic thiol-specific antioxidant (TSA), the *L. major* stress-inducible protein-1 (LmSTI1) and the *L. braziliensis* elongation and initiation factor (LeIF).[55] Initial immunisation trials in mice demonstrated that Leish-111f was able to protect mice against *L. major* and *L. amazonensis* infection.[56] There is some evidence that the Leish-111f vaccine can also induce partial protection against visceral leishmaniasis in animal models,[57] however, Leish-111f failed to protect dogs against infection and did not prevent disease development in a recent Phase III trial in dogs.[58] A slightly improved version of the original construct, Leish-110f, has also been tested in dogs as a therapeutic vaccine in combination with chemotherapy and led to reduced number of deaths and higher survival probability.[59] Human Phase I and II clinical trials (safety and immunogenicity) of Leish-111f have been completed over the past few years in Brazil, Peru and Columbia, and Phase I trial has been conducted in India (http://clinicaltrials.gov).

*Leishmania* parasites are transmitted from one host to another during the sand fly bite as a suspension in sand fly saliva. Sand fly saliva contains an array of molecules able to interfere with the host immune responses,[60] therefore, immunity against saliva components may indirectly enhance anti-leishmanial immunity. Prior exposure of mice to bites of uninfected sand flies conferred protection from *L. major* infection.[61] Immunization with molecules present in saliva, such as maxadilan[62] or a 15 kDa protein, SP15[63] also induced protection against cutaneous leishmaniasis. More recently, it has been shown that vector salivary proteins, in particular LJM19, protect hamster from VL,[64] and immunization of dogs with salivary antigens led to the development of high IgG2 antibody levels and significant IFN-γ production.[65]

## LIVE - ATTENUATED *LEISHMANIA* AS AN ALTERNATIVE FOR VACCINE DEVELOPMENT

The subunit vaccines tested so far did not lead to development of long-term immunity, and the whole cell killed vaccines have performed disappointingly in the field trials. Thus, the live-attenuated vaccine provides an appealing alternative. By mimicking the natural infection, live-attenuated parasites can deliver a complete spectrum of antigens to the antigen presenting cells, in principle leading to a better immune response that results in a better protective outcome than that observed following immunization with a subunit vaccine.

Avirulent microorganisms can be generated by a defined genetic alteration, eliminating the risk of parasite reversion to the virulent phenotype. Only a handful of attenuated strains have been tested so far with various outcomes, and the live-attenuated, antileishmanial vaccine is still at its early stages of development. Vaccination with dihydrofolate reductase thymidylate synthase (*dhfr-ts*) knockout parasites led to protection in a mouse model,[66] but failed to protect monkeys against an infectious challenge.[67] Deletion of cysteine proteinases in *L. mexicana* led to an attenuated strain capable of triggering partial protection against challenge in animal models.[68,69] These moderately encouraging results were thought to be due to rapid elimination of parasites by the host, since knockout parasites were not persistent. Conversely, *L. major* parasites lacking the *lpg2* gene persisted in mice without pathology and were able to confer protection against infection.[70] However, over time these mutants regained their ability to cause disease in the absence of the *lpg2* gene through an unknown compensatory mechanism,[71] suggesting that persistence may not be a desirable feature of a live-attenuated vaccine. Recently, *L. donovani* centrin null mutants (LdCEN^-/-^) have been reported to have selective growth arrest in the amastigote stage of development, but were viable in culture as promastigotes.[72] Centrin is a calcium-binding cytoskeletal protein involved in the duplication of centrosomes in higher eukaryotes. These mutants were unable to survive *in vitro* in human macrophages and animals vaccinated with LdCEN^-/-^ mutants were protected against homologous as well as heterologous challenge.[73] Our group has recently demonstrated that *L. major* phosphomannomutase (PMM) deficient mutants were able to protect susceptible mice against infection via an increased magnitude of T cell responses and suppression of IL-10 and IL-13 production early during infection.[74] These parasites are viable *in vitro*, but do not survive in macrophages or *in vivo* in mice, similarly to LdCEN^-/-^ parasites. Paradoxically, human PMM2-complemented ΔPMM parasites showed restored glycoconjugate biosynthesis, but remained avirulent *in vivo*, behavior reminiscent of ΔPMM parasites. Unexpectedly, the complementation with PMM2 led to the loss of protective capacity of ΔPMM parasites.[75] We have suggested that the glycoconjugates expressed by the add-back parasites were sufficient to interfere with the dendritic cell and macrophage functions upon priming, and subsequently led to decreased numbers of primed T cells or impaired T cell activation upon challenge. Recently, our speculations have been experimentally confirmed by Liu *et al*.[76] who showed that leishmanial glycoconjugates might prevent DC antigen presentation capacity affecting their Th1 cell inducing capabilities. Another example of an attenuated vaccine that showed protective efficacy is *L. infantum SIR2* single knockout strain,[77] however, the inherent problem of this strain is the presence of the second *SIR2* allele making reversion to virulence a likely occurrence. An interesting alternative to genetically attenuated strains is the use of non-pathogenic species such as *L. tarentolae* as live vaccines, an approach that has been proven successful in mice against VL.[78]

## IMPLICATIONS FOR VACCINE DESIGN

Vaccination is by far the most cost effective means of control of infectious diseases. Several vaccines have proved very efficient in controlling infections, and have led to complete eradication of diseases such as smallpox, or almost complete eradication of polio with just over 1500 cases recorded last year (www.polioeradication.org). Nevertheless, a number of important infectious diseases such as malaria, hepatitis C, HIV/AIDS or leishmaniasis continue to escape attempts to develop effective vaccines against them. That leishmaniasis ought to be controllable by vaccination seems indisputable in view of the body of experimental evidence. Yet, no vaccine is currently on the market despite much effort. Therefore, the question arises - what is the major problem in the antileishmanial vaccine development process?

Socio-economic and financial aspects notwithstanding, there are still unresolved scientific issues. Is parasite persistence required to maintain antileishmanial immunity in humans? Parasite persistence following infection has been demonstrated in experimental mouse model, but unlike murine studies, factors involved in parasite persistence in humans are not known.[79] Some studies show that complete parasite clearance leads to loss of immunity,[80] which might suggest that antileishmanial immunological memory does not develop and continuous antigenic presence is needed for protection. On the other hand, it has been shown that the maintenance of central memory T cells does not require parasite persistence[81] and vaccination with non-persistent, attenuated strains such as LdCEN^-/-^ or ΔPMM leads to long term protection. However, these observations have been derived from a murine model and at present it is impossible to extrapolate these findings to humans. The immune response to *Leishmania* is very complex and it appears that no vaccine exists against the disease due to our limited understanding of the T cell determinants needed for long-lasting protective immunity. We need to dissect the factors that contribute to antileishmanial immunity if we are to rationally design vaccines or immunotherapy protocols.

Recent insights into antileishmanial immunity offered possible explanations for the failure of the first generation vaccines in the field and have important implications for the vaccination strategies against leishmaniasis. Peters *et al.*[82] have demonstrated that sand fly transmission of parasites abrogates vaccine-induced protective immunity. While mice vaccinated with killed parasites were refractory to a needle challenge, they were susceptible to the sand fly inoculum implying that the responses in vaccinated mice required for protection were either not generated or not maintained. On the other hand, mice that healed the primary lesion were protected against sand fly challenge, and the rapidity of the response suggested that the protective response was not derived from the central memory, but rather from an effector pool of T cells that could have been maintained by the persistent parasites. These data provide a rationale for the inclusion of sand fly saliva components, which are specific to natural infection. In addition, it has been demonstrated that inoculation of killed parasites into immune mice leads to a loss of infection induced immunity.[83] This situation might be analogous to that observed in endemic areas, where many individuals with subclinical leishmaniasis were vaccinated with killed vaccines, which subsequently led to a loss of naturally acquired immunity and vaccination failure. Although this hypothesis has not been proven in humans, it clearly demonstrates that there are still many unknown factors that need to be unraveled before a successful vaccine becomes a reality.

Selection of vaccine candidates has continued to be an extremely difficult problem. As outlined in this review, a plethora of antigens have been evaluated with mixed success depending on the formulation and the animal model used for testing. However, complete protection has not been achieved so far and immunization has usually led only to partial protection. In addition, the opinions on the nature of the vaccine have been divided. Some argue that a vaccine against leishmaniasis should be molecularly defined, while others argue for a live attenuated vaccine. If the live attenuated vaccine is considered, efficacy may need to be balanced by safety.

One of the major problems facing a vaccine against cutaneous leishmaniasis, for example, is the fact that despite causing cutaneous disease, the old and new world parasites, *L. major* and *L. mexicana/L. amazonensis*, respectively, are markedly different.[84] There are differences in virulence factors between these species as well as in the immune responses that they induce. For example, LPG is a virulence factor for *L. major*,[85] but not for *L. mexicana.*[86] During the *L. major* infection the protective role of Th1 responses has been established, but *L. amazonensis* is able to persist in the presence of Th1 responses, and causes minimal disease in the complete absence of T cells.[87] These findings highlight major, but poorly understood differences in the immunobiology of parasites that seemingly cause the same disease. These may have implications for the vaccine development process since anti-CL vaccine may have different requirements for the old world and new world leishmaniasis. Therefore, a vaccine against *L. major*-caused CL might not necessarily be effective against the new world spectrum of diseases including mucocutaneous and diffuse cutaneous forms. Yet another challenge for the vaccine is to obtain protection against VL even if it is efficacious against the different forms of CL.

## CONCLUDING REMARKS

Preventive vaccines are recognized as the best and most cost-effective protection measure against pathogens, and are saving millions of lives every year across the globe. *Leishmania* vaccine development has proven to be a difficult and challenging task, which is mostly hampered by inadequate knowledge of parasite pathogenesis and the complexity of immune responses needed for protection. It is highly unlikely that a successful antileishmanial vaccine will be based on a single antigen. Combination vaccines composed of multiple antigens and well-developed adjuvants, such as Leish-111f and MPL-SE, have the best chances to succeed. Additional clinical trials should soon provide important information on the potential use of this combination. Considering the poor protective efficacy of killed vaccines and difficulties in formulating a subunit vaccine, the use of live-attenuated strains represents a promising alternative.

At the moment, major safety concerns and manufacturing considerations place this type of anti-*Leishmania* vaccines in the distant future. Nevertheless, development of new genetic engineering technologies and “hit and run”[88] targeting strategies can alleviate current problems associated with live-attenuated vaccines. Our understanding of T cell determinants needed for long-lasting protective immunity, while still fragmentary, offers hope for development of new strategies for effective T cell vaccines. The main concerns are reliable correlates of immunity that need to be developed in order to evaluate vaccines, and the development of an efficient delivery systems and improved adjuvants. Given the rapid progress in the fields of parasite immunology and genetic engineering, a successful anti-*Leishmania* vaccine should be achievable sooner rather than later.
